# Switchable ultra-broadband terahertz wave absorption with VO_2_-based metasurface

**DOI:** 10.1038/s41598-022-04772-4

**Published:** 2022-02-15

**Authors:** Nanli Mou, Bing Tang, Jingzhou Li, Hongxing Dong, Long Zhang

**Affiliations:** 1 Hangzhou Institute for Advanced Study, University of Chinese Academy of Sciences, Hangzhou, 310024 China; 2 Key Laboratory of Materials for High-Power Laser, Shanghai Institute of Optics and Fine Mechanics, Chinese Academy of Sciences, Shanghai, 201800 China; 3 CAS Center for Excellence in Ultra-Intense Laser Science, Shanghai, 201800 China; 4 Department of Materials Science and Engineering, and Centre for Functional Photonics (CFP), City University of Hong Kong, Hong Kong, 999077 SAR China

**Keywords:** Applied optics, Optical materials and structures

## Abstract

Metamaterial absorbers (MMAs) offer a novel and flexible method to realize perfect absorption in specific frequencies, especially in the THz range. Despite the exotic abilities to manipulate light, most previously reported MMAs still suffer from limited bandwidth and tunability. Here we present a thermally switchable terahertz (THz) metasurface that exhibits ultra-broadband absorption and high-transmission characteristics at different ambient temperatures. Our simulations demonstrate that at room temperature the structure is highly transparent. When the ambient temperature reaches 358 K, the proposed design exhibits an ultra-broadband absorption from 0.398 to 1.356 THz with the absorptivity maintaining above 90% and the relative absorption bandwidth reaches up to 109.2%. The structure is demonstrated to be insensitive to the incident angle. Moreover, the bandwidth of such a structure can easily be expanded or reduced by cascading or removing the rings, providing high scalability in practical applications. Such a thermally switchable THz metasurface may have potential applications in various fields, such as optical switching, THz imaging, modulating and filtering.

## Introduction

Electromagnetic (EM) absorption plays a central role in many practical devices, such as solar photovoltaic cells, thermal emitters, sensors, detectors, camouflage devices, radiative cooling, etc.^1–4^. Although enormous efforts have been devoted to the practicality of absorbers, much work still remains to be done, such as decreasing the thickness of devices, and increasing the controllability of the absorption properties^3^. Over the past decades, metamaterials (MMs), a kind of artificial structural materials which can utilize resonance inside plasmonic or dielectric materials, have shown unprecedented abilities to manipulate EM waves^5,6^. Owing to their ability of tailoring effective electric permittivity and magnetic permeability independently, MMs exhibit many unusual EM properties that are difficult to achieve with traditional materials, such as negative refraction, super-resolution imaging, electromagnetic cloaking, etc.^7–9^. In 2008, Landy et al.^10^ reported a reflection-type MMA with a metal–insulator-metal (MIM) sandwiched structure. By matching electric permittivity and magnetic permeability, a MMA can be impedance-matched to free space, thus minimizing reflectivity. Since this invention, a large number of reflection-type metasurfaces have been proposed and demonstrated to achieve perfect EM-wave absorption with working frequencies ranging from microwave to optical regions^3^.

In particular, THz absorbers are an important type of MMA. The THz wave (0.1–10 THz) is of great interest to researchers due to its potential applications in security, biomedicine, and high-bit-rate communication. The key problem limiting the THz technology is the lack of natural materials that can directly interact with THz waves. Therefore, research on THz MMA is of great importance to fulfill the “THz gap”. Tao et al.^11^ first demonstrated a narrowband absorber in the THz band. Thereafter, many MMAs were reported for the THz region^12–14^. However, for practical use, there are still some problems that need to be solved, including the narrow working bandwidth, which results from the intrinsic feature of resonant absorption, and lack of flexibility due to the fixed refraction index of constituent materials.

Many efforts have been devoted to broaden the bandwidth and make the MMAs tunable^15–19^. For instance, in order to expand the bandwidth, methods such as incorporating multiple resonant modes on a plane or in multiple layers^20–25^, and lowering the Q-factor in the resonant system by using high-loss metals^26–28^ have been extensively studied. On the other hand, tunable MMs based on active materials such as graphene^29–33^, or phase-changing materials (PCMs)^34–42^ have also been widely investigated. In particular, vanadium dioxide (VO_2_)-based MMAs exhibit unique properties thanks to the insulator-to-metal transition (IMT) feature of VO_2_. Many VO_2_-based dynamic MMAs were reported^43–47^, which typically consist of VO_2_ film that offers a variable dielectric environment, or differently patterned VO_2_ to generate localized surface plasmonic resonances (LSPRs). Despite the intense researches on dynamic broadband absorbers, most of the previous works still suffer from limited absorption bandwidth and/or complicated/multi-shaped resonators. A new design strategy to achieve switchable, ultra-broadband and highly-efficient absorbers with high scalability are highly demanded.

In this paper, we propose a thermally switchable THz metasurface that can work as an ultra-broadband MMA and a high-transmission structure at 358 K and 298 K, respectively, based on the IMT feature of VO_2_. Our simulations demonstrate that at 358 K, the proposed design exhibits an ultra-broadband absorption ranging from 0.398 to 1.356 THz, with the absorptivity above 90% and the relative absorption bandwidth (RAB) reaches up to 109.2%. Electric field distributions at the absorption peaks show that multiple hybrid plasmonic resonant modes contribute collectively to the observed ultra-broadband absorption bandwidth. A SiO_2_ layer is designed on top of the VO_2_-based MIM configuration to improve the impedance match via the cavity resonance, which provides a new mechanism to tune and optimize the absorption performance. Moreover, the bandwidth of such a structure can easily be expanded or shrunk by cascading or removing the rings, providing high scalability for practical applications. At 298 K, the VO_2_ behaves as a transparent dielectric, the interaction between the VO_2_ structure and incident waves is relatively weak, thus the structure is highly transparent. Compared with most of the previous works (see Supporting information Table S1), we realized an ultra-broadband MMA with much broader absorption bandwidth. Apart from the superposing of absorption spectra resulted from different resonators, the hybridization effect of ring-shaped resonators would push the resonant modes to higher/lower energy and thus further enlarge the absorption bandwidth^48^. Such a thermally switchable THz metasurface may have potential applications in various fields, such as optical switching, THz imaging, modulating and filtering.

## Design and methods

A typical MMA configuration is generally composed of metal resonator/dielectric/metal mirror sandwich layers. By optimizing the geometric size and arrangement, we can tune the impedance of the structure to match with the free space, thus limiting the reflectance. Furthermore, the bottom metal mirror prevents light transmission, thus the specific incident light can be confined in the MMA until it is completely absorbed. Moreover, in order to achieve efficient broadband absorption, two key points of structural design strategy are: firstly, more resonators with diffierent structures or sizes, which can generate adjacent absorption spectra and further overlap to form broadband absorption, are combined in limited periodicity; secondly, proper ohmic loss can be introduced to decrease the Q factor of absorption spectrum, which can further expand the absorption bandwidth and flatten the absorption spectra.

**Figure 1:**
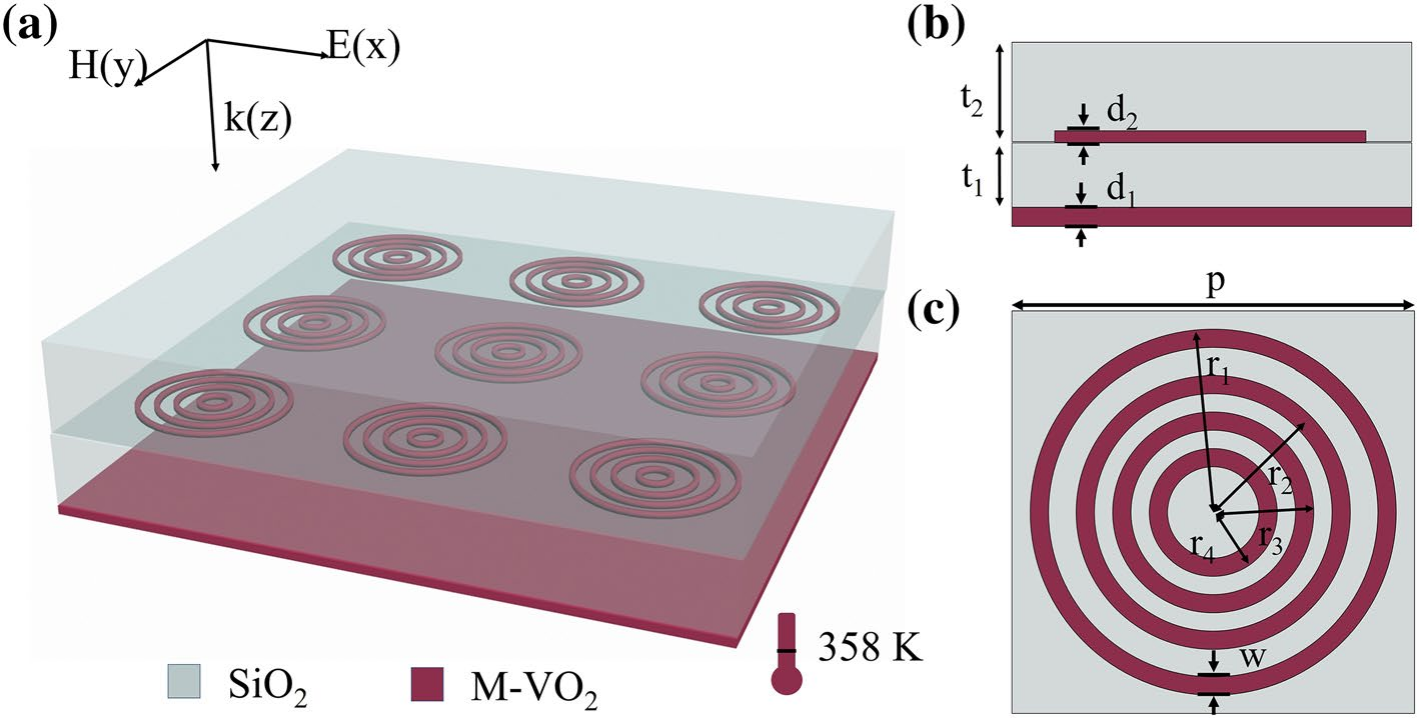
(**a**) Schematic diagram of the ultra-broadband adjustable metamaterial absorbing structure based on VO_2_. Four-ring nested VO_2_ periodic array structure with thickness of 0.5 μm is embedded between two layers of SiO_2_. Bottom layer is 1 μm-thick VO_2_ film. At 358 K, VO_2_ transforms into metal state, and the structure exhibits THz broadband absorption characteristics. (**b**) Side-view and (**c**) top-view of unit cell. Geometric parameters are p = 90 μm, r_1_ = 42 μm, r_2_ = 34 μm, r_3_ = 25 μm, r_4_ = 19 μm, t_1_ = 22 μm, t_2_ = 39 μm, d_1_ = 1 μm, d_2_ = 0.5 μm, and w = 2 μm, respectively.

The simulation works are carried out using finite element method (FEM) with commercially available software COMSOL Multiphysics 5.5. A unit cell of the proposed absorber is simulated using periodic boundary condition along the in-plane x- and y- axes and perfectly matched layers along the propagation direction (z-axis). Inspired by the above discussions, here we propose an ultra-broadband THz MMA based on multi-ring metallic VO_2_ resonator array (MMVA), as shown in Fig. 1. Four 0.5 µm-thick concentric VO_2_ rings are imbedded in two dielectric layers, and 1 µm-thick VO_2_ continuous film is deposited at the bottom of the structure. Figure 1b, c exhibit the side view and top view of an unit cell. Due to the IMT phase-change feature of VO_2_, the MMVA layer exhibits the same behavior as metal resonators when the ambient temperature is above 358 K, and can strongly react with the incident wave, and generate an LSPR mode, which will couple with the bottom metallic VO_2_ film. On the other hand, at room temperature, VO_2_ behaves as a transparent dielectric material, resulting in a relatively weak interaction between the VO_2_ structure and incident light. Therefore, a temperature-switchable THz metasurface is achieved utilizing the IMT characteristics of VO_2_.

The dielectric permittivity of VO_2_ in THz range can be described by the Drude mode^49,50^ ${\upvarepsilon }\left( {\omega } \right) = {\upvarepsilon }\left( {\omega } \right) = {\upvarepsilon }_{\infty }  - {\upomega }_{{\text{p}}}^{2} /({\upomega }^{2}  + {\text{i}}{\upgamma \upomega })$, where ${\upvarepsilon }_{\infty } = 12$ is the permittivity at high frequency, ${{ \upomega }}_{{\text{p}}} \left( {\upsigma } \right)$ is the conductivity-dependent plasmon frequency, and ${\upgamma } = 5.75 \times 10^{13 }  \,{\text{rad/s}}$ stands for the collision frequency^36,51^. In addition, ${\upomega }_{{\text{p}}}^{2} \left( {\upsigma } \right)$ and ${\upsigma }$ are proportional to the free carrier density. At a specific conductivity, $\sigma^{\prime}$, the plasmon frequency can approximately be defined by ${\upomega }_{{\text{p}}} \left( {{{\upsigma^{\prime}}}} \right) = \left( {{{\upsigma^{\prime}}}/{\upsigma }_{0} } \right){\upomega }_{{\text{p}}}^{2} \left( {{\upsigma }_{0} } \right)$, where ${\upsigma }_{0} = 3 \times 10^{5} {\text{ S}}/{\text{m}}$, and ${\upomega }_{{\text{p}}} \left( {{\upsigma }_{0} } \right) = 1.4 \times 10^{15} {\text{ rad}}/{\text{s}}$. The conductivity of VO_2_ film in the fully insulating and the metallic states, which correspond to 298 K (room temperature) and 358 K, are assumed to be $\sigma  = 200\;{\text{S/m}}$ and $200000\;{\text{S/m}}$, respectively^43,47^. SiO_2_ is modeled as lossless dielectric material with permittivity $\varepsilon = 3.8$ ^52,53^.The whole structure is considered to be placed on an infinite SiO_2_ substrate to avoid the Fabry–Perot resonance at room temperature.

## Results and discussions

**Figure 2:**
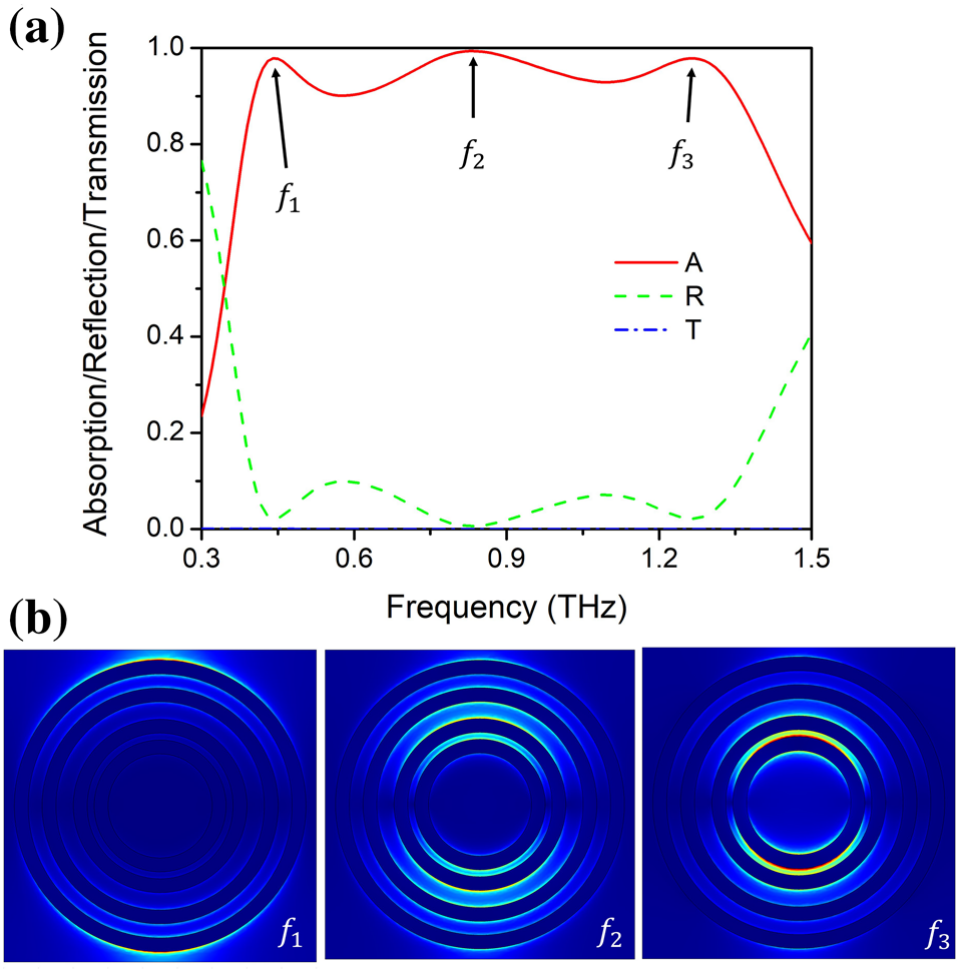
(**a**) Absorption spectra of ultra-broadband absorption structure at 358 K (VO_2_ conductivity of 200,000 S/m). Red solid line (A) represents absorption rate, green dotted line (R) represents reflectivity, and blue dotted line (T) stands for transmittance. (**b**) Distribution of electric field at three peak locations of ultra-broadband absorption spectrum.

We performed finite element method (FEM) numerical simulations to investigate the proposed design. Firstly, we considered the high temperature (358 K) condition, where VO_2_ is at the metallic state. As shown in Fig. 2, we calculated the reflection, transmission, and absorption spectra of the proposed design at 358 K. It is clear that in this working mode, the transmission spectrum values (blue dotted line) from 0.3 to 1.5 THz are almost 0, which demonstrates that a 1 µm-thick metallic state VO_2_ (M-VO_2_) film can effectively cut off transmission in this frequency range. Furthermore, according to the reflection spectrum (green dotted line), such a M-VO_2_ configuration can effectively tune the impedance of the structure to match with the free space from 0.398 to 1.356 THz, thus leading to the reflectivity lower than 0.1 in this range. The absorption of the structure can be calculated as A(ω) = 1 − T(ω) − R(ω), and is shown in Fig. 2a (red solid line). Obviously, at 358 K the structure exhibits a highly efficient (above 90%) absorptivity ranging from 0.398 to 1.356 THz. At 0.83 THz, the absorptivity exceeds 99%, which is near perfect absorption. In particular, the calculated RAB, defined as ${\text{ RAB}} = 2 \times \left( {{\text{f}}_{{\text{h}}} - {\text{f}}_{{\text{l}}} } \right)/\left( {{\text{f}}_{{\text{h}}} + {\text{f}}_{{\text{l}}} } \right)$, where ${\text{f}}_{{\text{h}}}$ and ${\text{f}}_{{\text{l}}}$ are the highest and lowest frequencies with absorptivity in excess of 90%, respectively, reaches about 109.2%.

It is worth noting that only three absorption peaks can be identified, even though we adopted four metallic VO_2_ ring array as resonators. This can be explained by the fact that the absorption peaks do not necessarily correspond to the resonances, especially in such a complex structure with a broadband absorption superposed by multiple resonance modes, one absorption peak can be a result of the superposition of multiple resonant absorption modes with small Q-values. In Fig. 2b we show the electric field distribution at the three absorption peaks. For simplicity, we denote the four rings from the largest to the smallest as r_1_–r_4_. It is clear that at f_1_ the electric field concentrates mainly at the edge of r_1_, and partly at the edge of r_2_, which means that the absorption peak at f_1_ can be attributed to the resonance of r_1_ and r_2_. Similarly, the absorption peak at f_3_ is generated by the resonance of r_3_ and r_4_. Moreover, at f_2_, the electric field concentrates at the edge of all the rings, which means that the absorption peak at f_2_ resulted from complex superposition and coupling effects of the four metallic VO_2_ rings.

**Figure 3:**
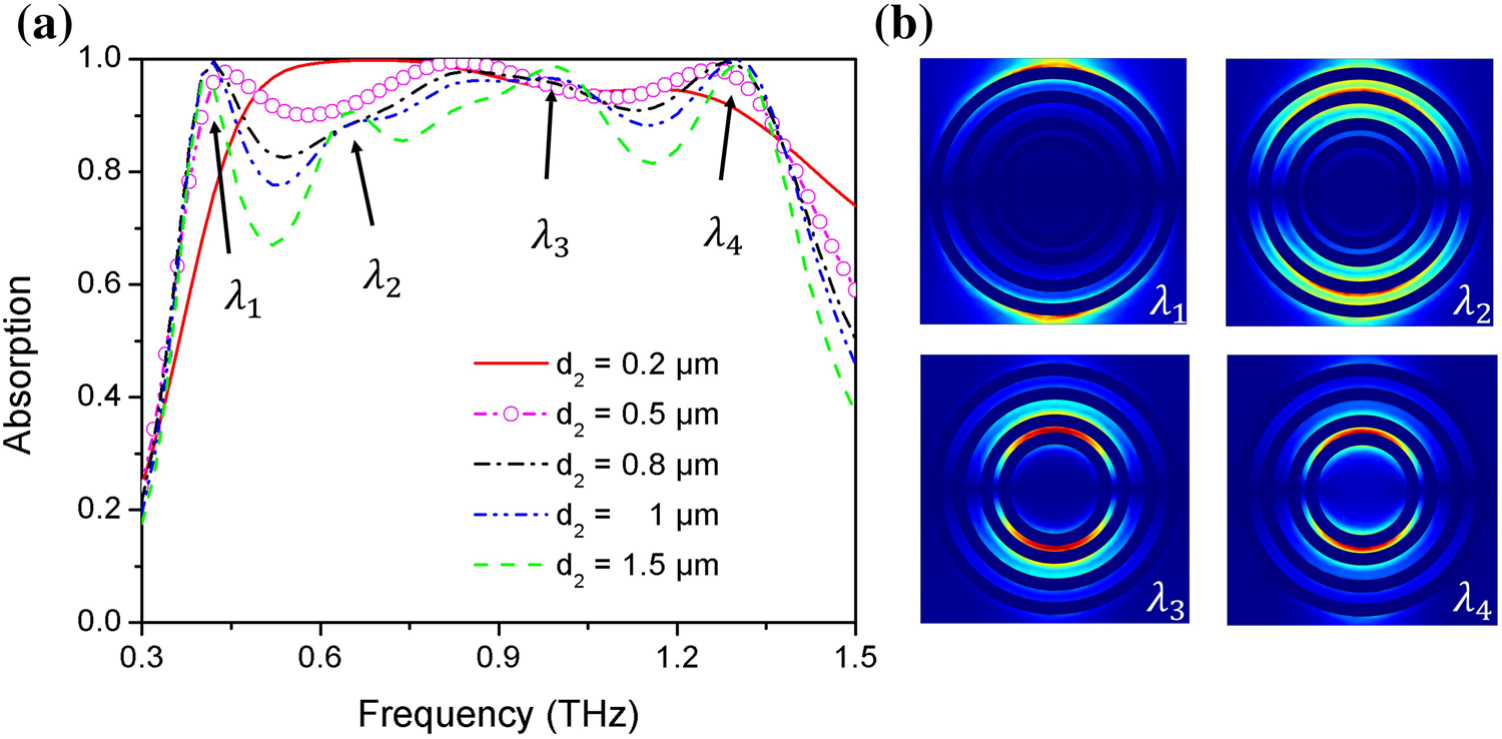
(**a**) Absorption spectra as a function of thickness of VO_2_ rings. (**b**) Electric field distributions at the four absorption peaks in the MMVA layer.

Due to the finite conductivity of metallic-state VO_2_, the electromagnetic responses of the absorber are sensitive to the thickness of MMVA. As shown in Fig. 3a, we calculated the absorption as a function of the thickness of MMVA d_2_. It can be seen that, at larger thickness, for example d_2_ = 1.5  µm, four absorption peaks can clearly be observed. Electric field distributions at the four absorption peaks in the MMVA layer are shown in Fig. 3b. Notably, the four absorption peaks correspond to the strong electric field concentration of rings with different radii. When d_2_ decreases, Q-factors of each absorption peaks decrease, resulting in an increase of the dips between absorption peaks, i.e., flattened the top. At the same time, the absorption bandwidth also decreases. When d_2_ = 0.5  µm, the dips between absorption peaks are increased to above 90%, and highly-efficient ultra-broadband absorption is achieved. When d_2_ decrease further to 0.2 μm, which is much smaller than the skin depth of metallic-state VO_2_ (about 1  µm at 1 THz) in this frequency range, the absorption bandwidth exhibits a significant decrease. This can be explained by that the decrease of d_2_ can lead to an increase of loss, thus weakening the resonance which further decreases the Q factor of the absorption spectrum generated by each ring resonator. The corresponding absorption factor Q_a_ and radiative factor Q_r_ are changed accordingly, resulting in significant change in the broadband absorption spectrum^54^.

**Figure 4:**
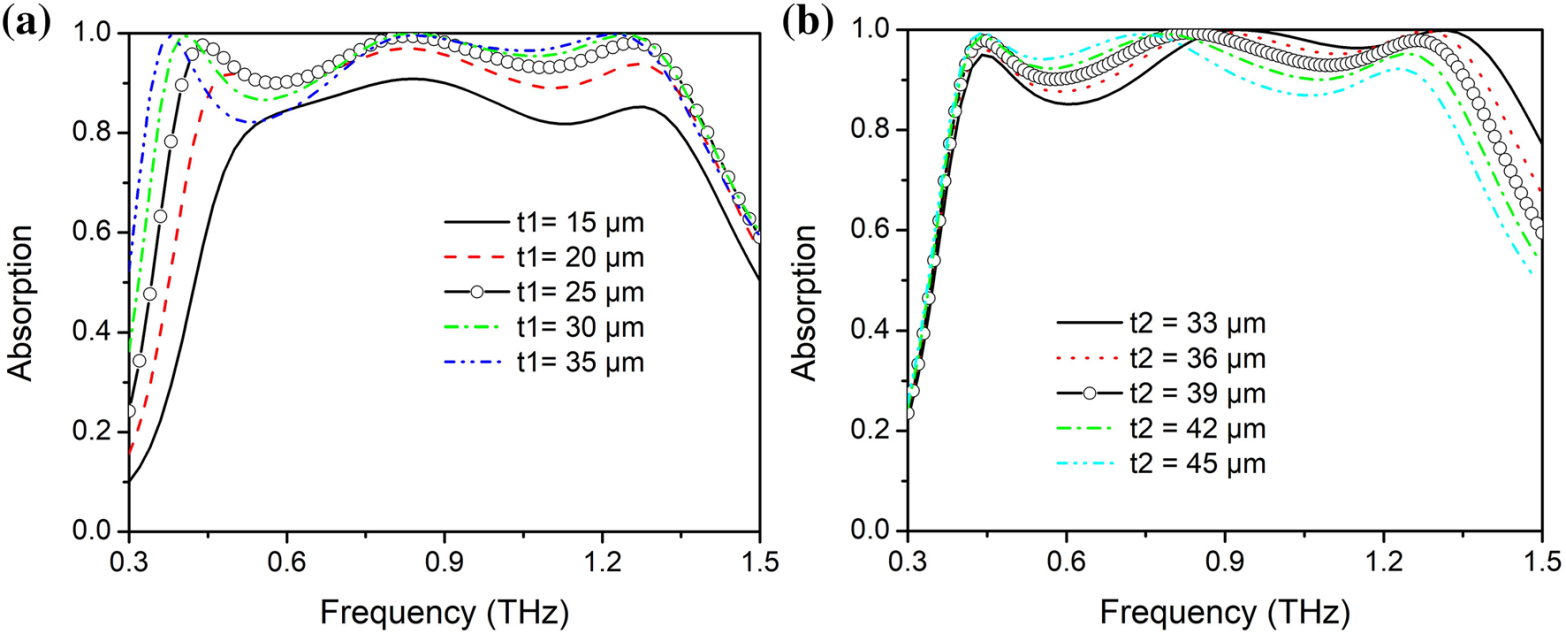
Absorption spectrum modification with thickness of SiO_2_ layers t_1_ (**a**) and t_2_ (**b**).

In order to improve the impedance matching and further optimize the broadband absorption performance, a layer of SiO_2_ is designed on top of the traditional MIM configuration, thus offering more freedom to shape the broadband absorption spectrum via tuning the thickness of two SiO_2_ layers. As shown in Fig. 4, the relationship between absorption spectra and the thickness of SiO_2_ layers t_1_ and t_2_ was analyzed. The thickness of lower SiO_2_ layer t_1_ can greatly influence the coupling effect between the MMVA and the bottom VO_2_ film, which can change the impedance condition and absorption performance of the structure obviously. As depicted in Fig. 4a, with an increase in t_1_, the absorptivity of the proposed structure exhibits an overall increase first, but when it reaches 100%, a part of the broadband absorption begins to decrease. This can be explained by the fact that with the increase in t_1_, each absorber based on a single VO_2_ ring will first exhibit an increase, and then a decrease, depending on the proximity to the impedance matching condition (IMC). Therefore, when t_1_ increases from 15 to 20  µm, the absorption corresponding to each ring increases due to approaching the IMC, and results in an overall increase of the superposed broadband absorption spectrum. When t_1_ increases gradually from 20 to 35  µm, part of the resonances increase beyond the critical point of IMC while the others not, which results in a decrease of the average absorptivity. As shown in Fig. 4b, with the increase of the upper SiO_2_ thickness t_2_, the absorptivity of higher frequency band decreases, while the lower frequency band increases. Due to the larger refractive index of SiO_2_ compared to vacuum, the THz waves reflected from the MIM structure can be reflected at the SiO_2_-air interface and enter into the MIM structure again to be repeatedly absorbed. The two SiO_2_ layers can also be considered as a resonant cavity that confines incident light with the Fabry–Perot resonance. The increase of cavity length will lead to red-shifting of the resonant wavelength, which will result in an increased absorptivity in the lower frequency range and a decrease in the higher frequency range, respectively.

**Figure 5:**
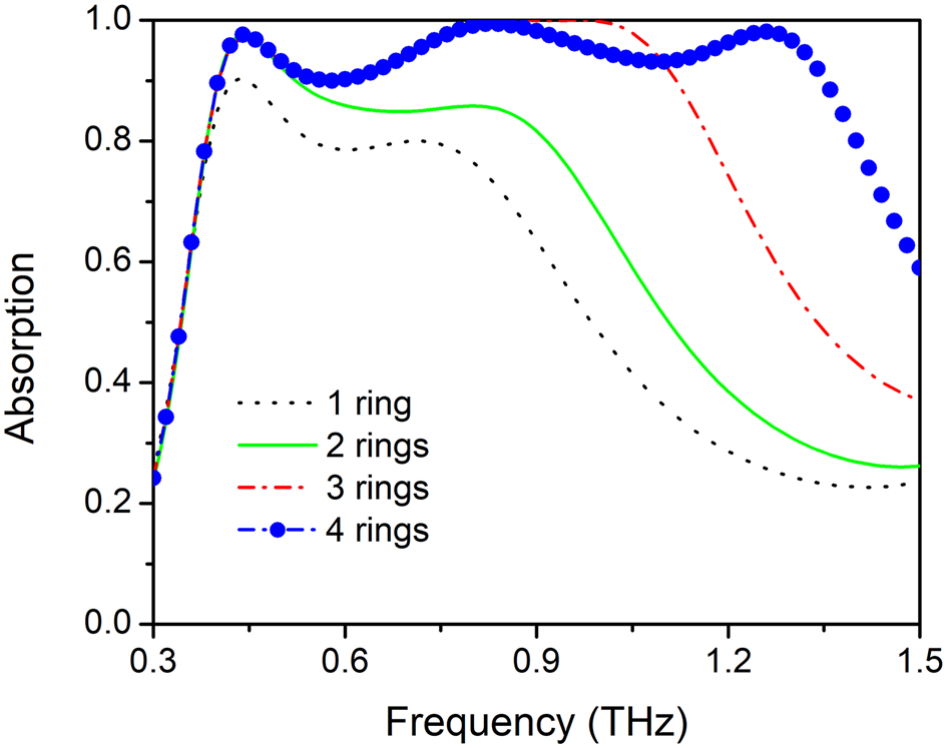
Absorption spectra with number of rings gradually increases from outside to inside.

Notably, such a broadband absorption design based on concentric-ring resonator offers high scalability to control the absorption bandwidth by removing or cascading VO_2_ rings. Figure 5 shows the absorption spectra with the number of rings gradually increasing from the outside to the inside. When there is only one outer ring with r_1_ = 42 μm, the broadband absorption spectrum ranges from 0.38 to 0.77 THz with absorptivity in excess of 78.5%. When the second ring is added, the broadband absorption bandwidth increases to the range from 0.38 to 0.85 THz with absorptivity larger than 85%. The broadband absorptivity is relatively small, because the thicknesses of the two SiO_2_ layers t_1_ and t_2_ are optimized for four-ring ultra-broadband absorption, and for each single ring it may be over or under the IMC. By changing t_1_ and t_2_, broadband absorption absorptivity above 90% may also be achieved under this condition. Increasing the number of rings further, the absorption bandwidth also shows a clear increase. When there are three rings in the MMVA, the absorption bandwidth increases to the range from 0.398 to 1.125 THz (with the absorptivity in excess of 90%), the lower frequency range absorption is almost the same as that in the two ring-based absorbers, while in the higher frequency ranging from 0.84 to 0.99 THz, the absorptivity reaches up to 99.9%, with a near perfect absorption bandwidth of approximately 16.4%. When there are four periodic VO_2_ rings in MMVA layer, as discussed before, the absorption bandwidth increases further to 0.398~1.356 THz with the absorptivity above 90% and RAB of approximately 109.2%.

**Figure 6:**
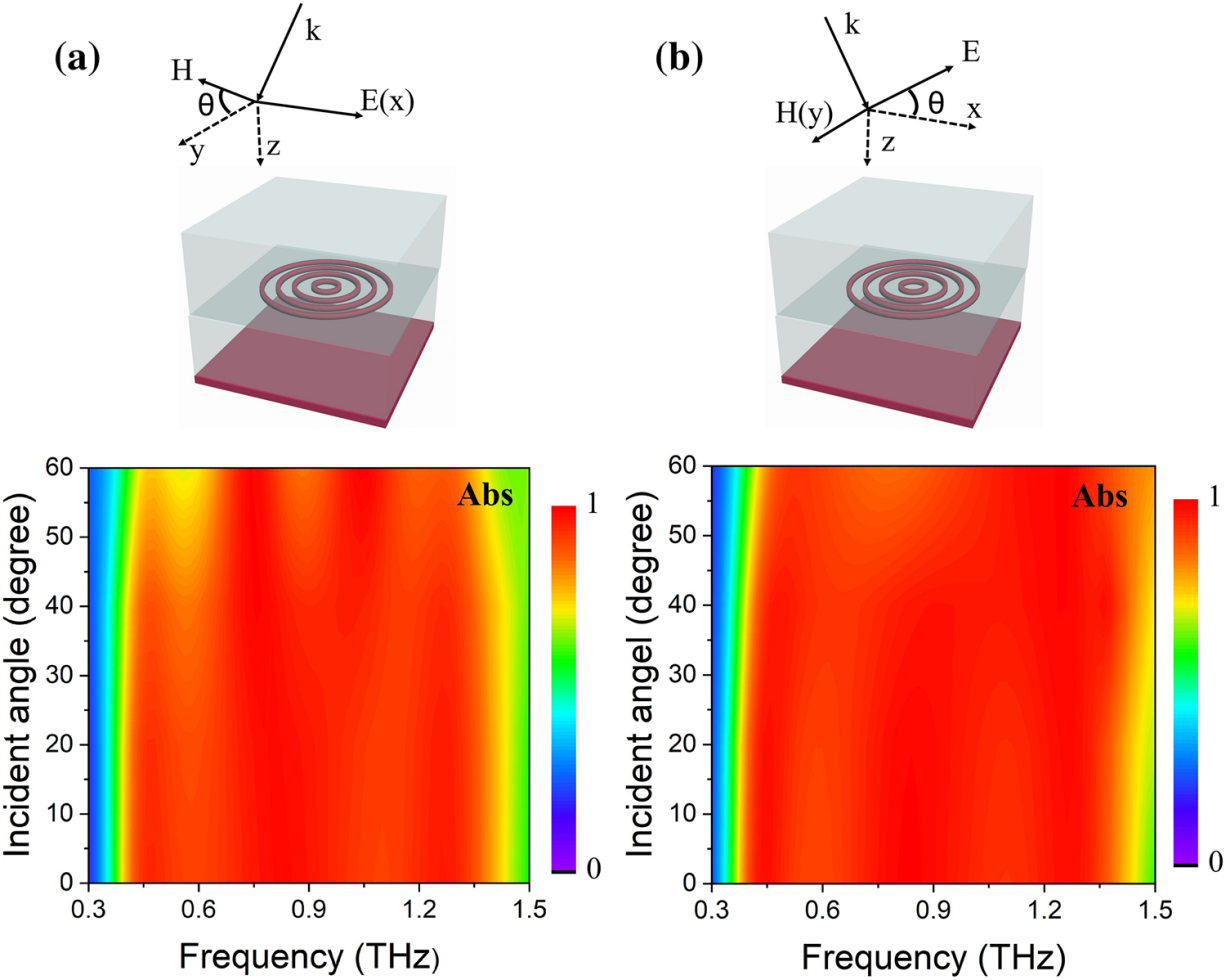
Absorption (Abs) spectra in (**a**) TE and (**b**) TM polarization states as function of incident angle.

In most practical applications, there is a requirement that the broadband absorption spectrum be relatively insensitive to the incident angle. Thus, we calculated the performance of the structure for incident angles from 0 to 60° and different polarization states when VO_2_ is in the metallic phase. Figure 6 presents the absorption maps as a function of the incident angle in transverse-electric (TE) and transverse-magnetic (TM) polarization states (the electric/magnetic field remains parallel to the x/y axis, as shown in the Fig. 6). It is worth noting that under both polarization states the structure maintains absorptivity larger than 80%, when the incident angle is lower than 40°. If the incident angle is further increased from 40° to 60°, the TE state condition exhibits an obvious decrease in absorption around 0.6 THz, but absorptivity remains larger than 70% at the incident angle of 60°. The obvious decrease in absorptivity may be attributed to the reverse current generated in the metallic VO_2_ rings due to the increase of magnetic component. On the other hand, at the TM polarization state the absorptivity remains larger than 80%, even when the incident angle reaches 60°. Moreover, we calculated the absorption spectra with the fluctuations of material parameters and geometrical dimensions (see session 3 in supporting information), and demonstrated the structure with excellent tolerance for experiment errors.

**Figure 7:**
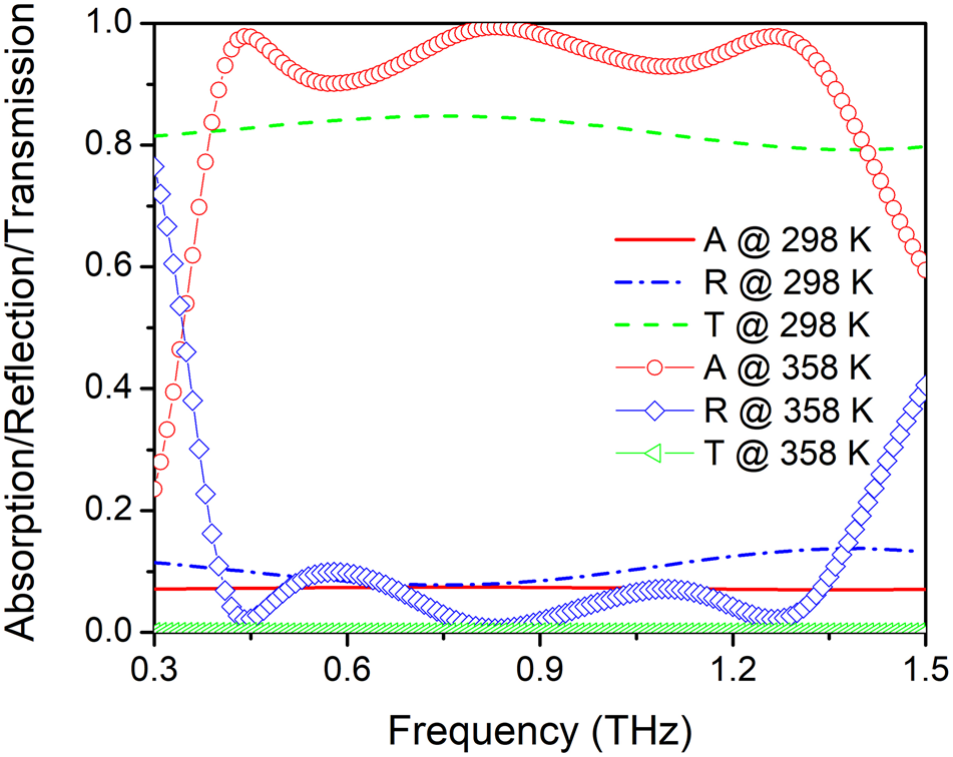
Transmission, reflection, and absorption spectra of structure at high temperature (358 K, VO_2_ conductivity of 200,000 S/m) and at room temperature (VO_2_ conductivity of 200 S/m). Red line (A) represents the absorption rate, blue line (R) represents the reflectivity, and green line (T) stand for the transmittance.

When the ambient temperature drops to 298 K (room temperature), corresponding to the conductivity of VO_2_ of 200 S/m, VO_2_ is in the dielectric-phase with a high refractive index. We have calculated the transmission, reflection, and absorption spectra of the structure at a high temperature (358 K, VO_2_ conductivity of 200,000 S/m) and room temperature (VO_2_ conductivity of 200 S/m), and show them in Fig. 7. It is noted that the proposed structure exhibits two completely different functions at different ambient temperatures. At 358 K, the device works as an ultra-broadband absorber with RAB of approximately 109.2% and absorptivity in excess of 90%. However, at 298 K the structure shows high transmission of approximately 80%, and the absorptivity drops to about 7% at the same time. Thus, utilizing the IMT characteristics of VO_2_, a switchable multifunction THz metasurface is achieved, which can work as an ultra-broadband absorber and a highly-transparent material. Moreover, during the IMT process, there are many intermediate states of VO_2_ with different conductivities. As shown in the Fig. S1 of supporting information, we calculated the absorption, transmission, reflection spectra with the increasing of VO_2_ conductivity from 200 to 200 000 S/m, the absorptivity in the range of 0.398~1.356 THz increase from below 10% to above 90% gradually and the transmittance decrease from above 80% to near 0. Since the intermediate states of VO_2_ are dependent on the heating temperature, such a phenomenon can be used as a temperature sensor or thermally controllable multilevel ultra-broadband absorber.

## Conclusions

We have proposed a thermally switchable THz metasurface that exhibits two totally different work modes at room temperature and high temperature on the basis of the IMT characteristic of VO_2_. At 358 K, VO_2_ exhibits metallic-phase characteristics, and can strongly interact with the incident THz waves. By superposing the resonance-induced absorption of the metallic-phase VO_2_-ring based absorbers, an ultra-broadband absorption ranging from 0.398 to 1.356 THz with absorptivity in excess of 90% and the RAB reaches up to 109.2%. Electric field distribution at the absorption peaks shows that multiple hybrid plasmonic resonant modes collectively contribute to the observed ultra-broadband absorption. At 298 K, VO_2_ exhibits the characteristics of a transparent dielectric. In this case the interaction between the VO_2_ structure and incident waves is relatively weak, thus the structure is highly transparent (approximately 80% transmission). Herein, the proposed thermally switchable THz metasurface may have potential applications in various fields, such as optical switching, THz imaging, modulating and filtering.

## Supplementary Information

Supplementary Information.
