# Supplementary material for: Switchable ultra-broadband terahertz wave absorption with VO2-based metasurface
Source: Sci Rep. 2022 Feb 15;12:2501. doi: 10.1038/s41598-022-04772-4 (PMC8847589; doi:10.1038/s41598-022-04772-4)
Supplement: Supplementary file 1 — Supplementary Information. [file 41598_2022_4772_MOESM1_ESM.docx]

Supporting Information

Switchable ultra-broadband terahertz wave absorption with VO_2_-based metasurface

**Nanli Mou^1,2^, Bing Tang^4^, Jingzhou Li^1^, Hongxing Dong^1,2,3*^, and Long Zhang^1,2,3*^**

^1^  Hangzhou Institute for Advanced Study, University of Chinese Academy of Sciences, Hangzhou 310024, China; m[ounanli@ucas.ac.cn](mailto:ounanli@ucas.ac.cn) (N. M.);

^2^ Key Laboratory of Materials for High-Power Laser, Shanghai Institute of Optics and Fine Mechanics, Chinese Academy of Sciences, Shanghai, 201800, [China; yaqiangzhang@siom.ac.cn(Y](mailto:China;%20yaqiangzhang@siom.ac.cn(Y). Z.);lzhang@siom.ac.cn(L.Z.);

^3^ Department of Materials Science and Engineering, and Centre for Functional Photonics (CFP), City University of Hong Kong, 999077 Hong Kong SAR, China; [tongsiom@mail.ustc.edu.cn(B](mailto:tongsiom@mail.ustc.edu.cn(B). T);

^4^ CAS Center for Excellence in Ultra-intense Laser Science, Shanghai 201800, China.

*****Correspondence: Hongxing Dong: [hongxingd@siom.ac.cn;](mailto:hongxingd@siom.ac.cn(H)

Long Zhang: [lzhang@siom.ac.cn](mailto:lzhang@siom.ac.cn)

**Session 1. Comparison of representative works on the topic of tunable/broadband THz MPAs.**

Table S1 Comparison of representative works on the topic of tunable/broadband THz MPAs.

| Work by | Operation bandwidth | Absorptivity | Maxmim Relative absorption bandwith | Active material |
| --- | --- | --- | --- | --- |
| *S. Wang, et al.* (2019)^1^ | 0.61~1.36 THz | >90% | (1.36-1.61)×2/(1.36+0.61) ~76.1% | VO_2_ |
| *H. Liu, et al.* (2019)^2^ | 1.27 ~2.94 THz | >90% | (2.94-1.27)×2/(2.94+1.27) ~79% | VO_2_ |
| *F. Ding, et al.* 2018^3^ | 0.56~1.232 THz | >90% | (1.232-0.56)×2/(1.232+0.56) ~74.7% | VO_2_ |
| *Z. Song, et al.* (2020)^4^ | 0.393 ~ 0.897 THz | >90% | (0.897-0.393)×2/(0.897+0.393)~78.1% | VO_2_ |
| *X. Kong, et al.* (2019)^5^ | 7.36~16.67 THz | >90% | (16.67-7.36)×2/(16.67+7.36) ~77.4% | VO_2_ |
| *Y. Zhang, et al.*  (2020)^6^ | 6.6~8.9 THz | >90% | (8.9-6.6)×2/(8.9+6.6)~29.7% | VO_2_ |
| *R. Dao, et al.*  (2019)^7^ | 1.63~3.86 THz | >90% | (3.86-1.63)×2/(3.86+1.63)~81.2% | VO_2_ |
| *Y. Liu, et al.*  (2021)^8^ | 0.78~1.16 THz | >90% | (1.16-0.78)×2/(1.16+0.78)~39.1% | STO  graphene |
| *J. Zhu, et al.*  (2021)^9^ | 1.1866~1.5783 THz | >90% | (1.5783-1.1866)×2/(1.5783+1.1866)~28.3% | graphene |
| **This work** | **0.398 ~1.356 THz** | **>90%** | **(1.356-0.398)×2/(1356+0.398)~109.2%** | **VO_2_** |

1. Wang, S. *et al.* Vanadium dioxide based broadband THz metamaterial absorbers with high tunability: simulation study. *Opt. Express* **27**, 19436 (2019).

2. Liu, H., Wang, Z., Li, L., Fan, Y. & Tao, Z. Vanadium dioxide-assisted broadband tunable terahertz metamaterial absorber. *Sci. Rep.* **9**, 5751 (2019).

3. Ding, F., Zhong, S. & Bozhevolnyi, S. I. Vanadium Dioxide Integrated Metasurfaces with Switchable Functionalities at Terahertz Frequencies. *Adv. Opt. Mater.* **6**, 1701204 (2018).

4. Song, Z., Chen, A. & Zhang, J. Terahertz switching between broadband absorption and narrowband absorption. *Opt. Express* **28**, 2037 (2020).

5. Kong, X., Dao, R. & Zhang, H. A Tunable Double-Decker Ultra-Broadband THz Absorber Based on a Phase Change Material. *Plasmonics* **14**, 1233–1241 (2019).

6. Zhang, Y. *et al.* Study on Temperature Adjustable Terahertz Metamaterial Absorber Based on Vanadium Dioxide. *IEEE Access* **8**, 85154–85161 (2020).

7. Dao, R., Kong, X., Zhang, H.-F. & Tian, X. A Tunable Ultra-Broadband Metamaterial Absorber with Multilayered Structure. *Plasmonics* **15**, 169–175 (2020).

8. Liu, Y., Huang, R. & Ouyang, Z. Numerical Investigation of Graphene and STO Based Tunable Terahertz Absorber with Switchable Bifunctionality of Broadband and Narrowband Absorption. *Nanomaterials* **11**, 2044 (2021).

9. Zhu, J., Wu, C. & Ren, Y. Broadband terahertz metamaterial absorber based on graphene resonators with perfect absorption. *Results Phys.* **26**, 104466 (2021).

**Session 2. The performances of the proposed design with the VO_2_ in different intermediate states.**


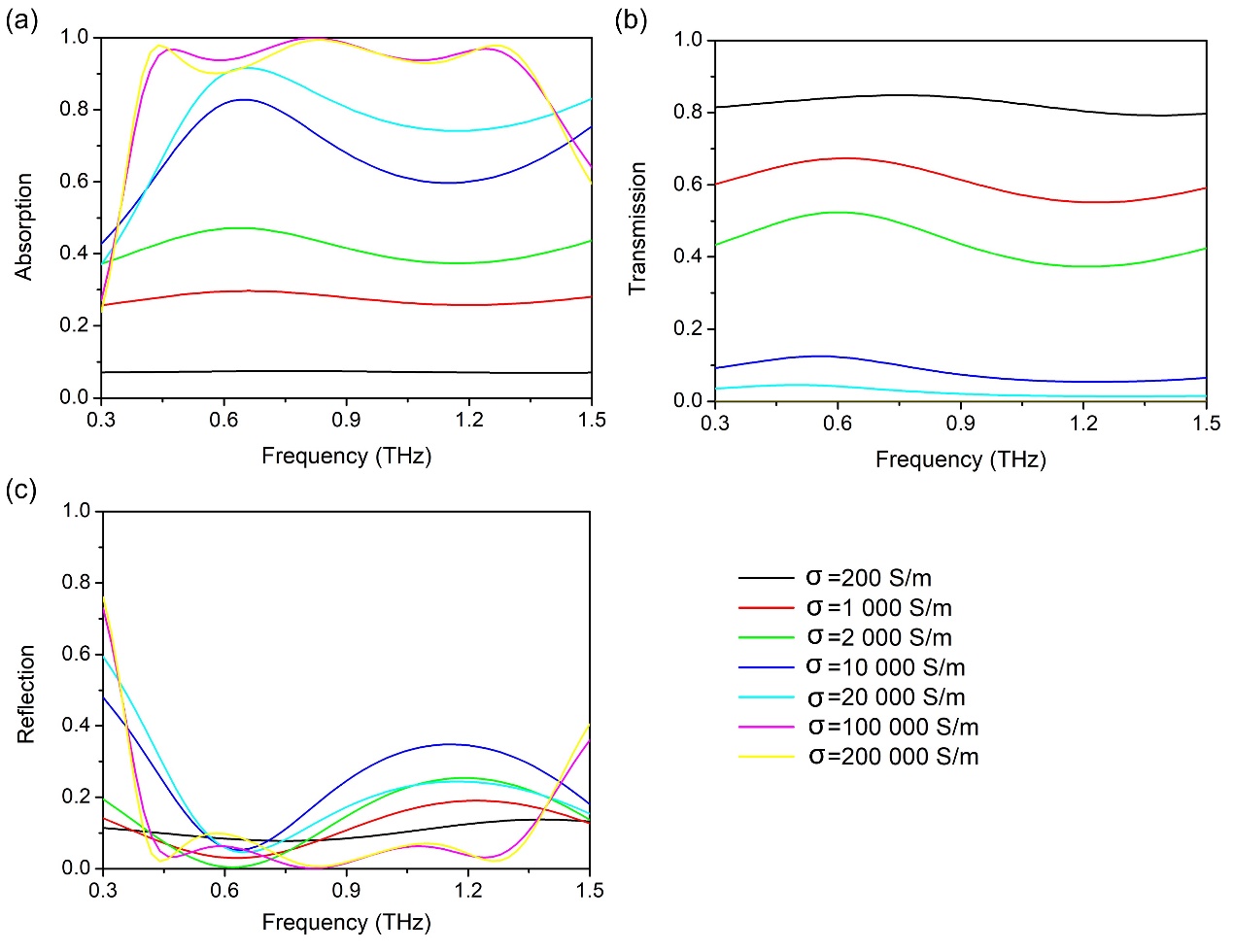


Figure S1. (a)Absorption, (b)transmission and (c)reflection spectra of the proposed structure with the conductivity of VO_2_ change from 200 S/m to 200 000 S/m.

**Session 3. The performances of the proposed design with the material optical constants and geometrical dimensions fluctuating from the optimized parameter.**


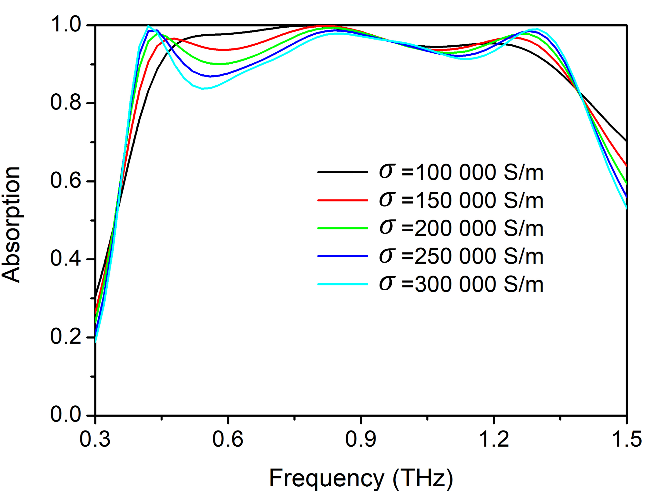


Figure S2. Absorption spectra of the proposed ultra-broadband MMA with the conductivity of metal-state VO_2_ fluctuating from the utilized material parameter , i.e. $\sigma_{0}=200 000 S/m$.


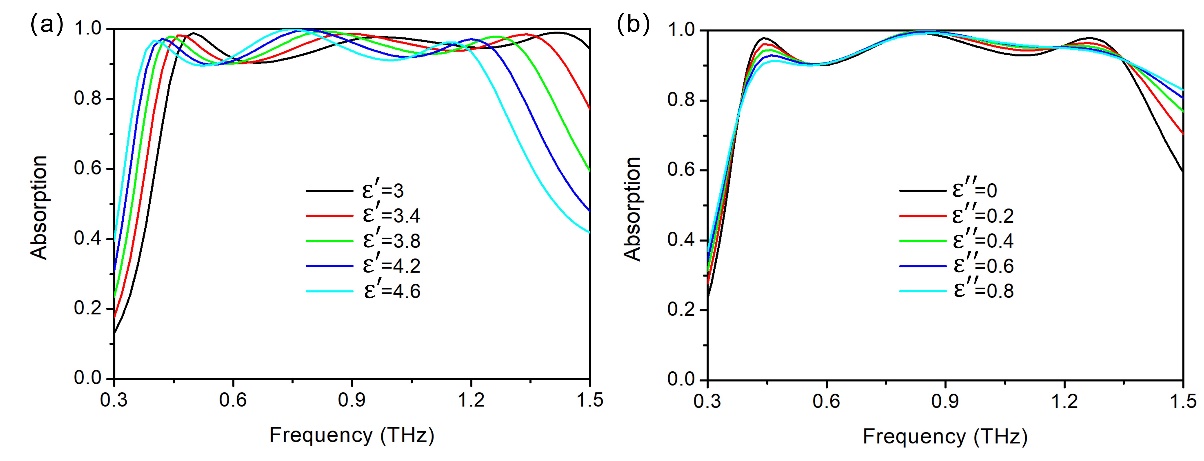


Figure S3. Absorption spectra of the proposed ultra-broadband MMA with the real and imaginary of the permittivity of SiO_2_ fluctuating from the utilized material parameter , i.e. $\varepsilon^{'}=3.8, \varepsilon''=0$.

**
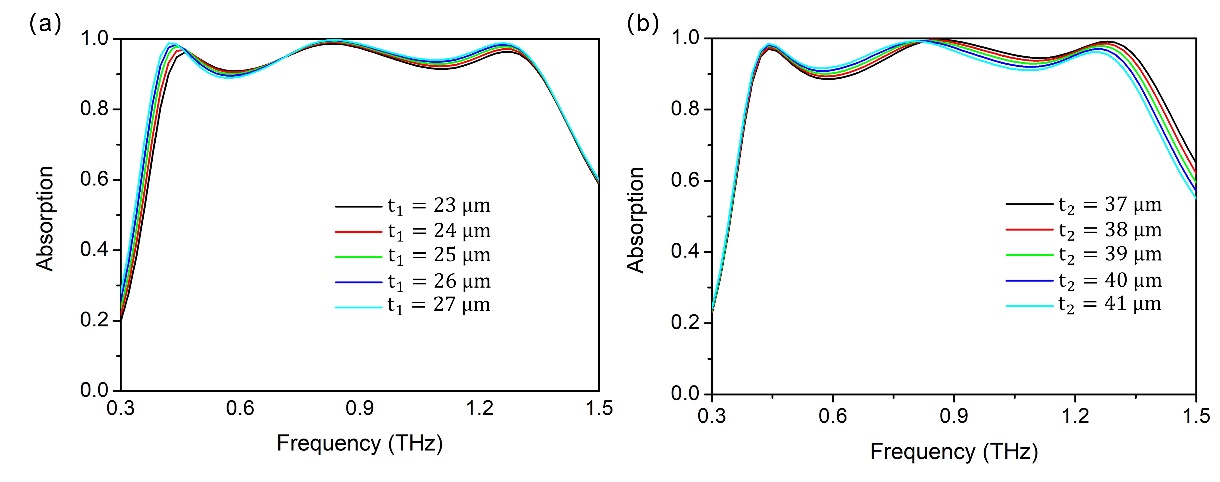
**

Figure S4. Absorption spectra of the proposed ultra-broadband MMA with the thickness of SiO_2_ (a) t_1_ and (b)t_2_ fluctuating from the designed thickness, i.e. t_1_=25 μm, t_2_=39 μm.


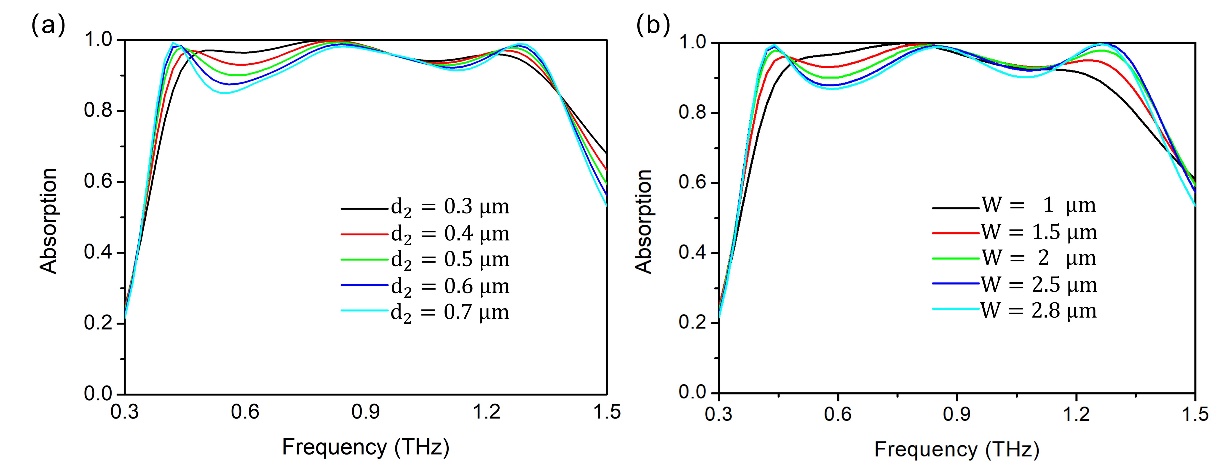


Figure S5. Absorption spectra of the proposed ultra-broadband MMA with the (a) thickness of VO_2_ rings d_2_ and (b) width of rings W fluctuating from the designed sizes, i.e. d_2_=0.5 μm, W=2 μm.
